# Supplementary material for: Enhancing student-centered walking environments on university campuses through street view imagery and machine learning
Source: PLoS One. 2025 Apr 9;20(4):e0321028. doi: 10.1371/journal.pone.0321028 (PMC11981197; doi:10.1371/journal.pone.0321028)
Supplement: S1 Table — (DOCX) [file pone.0321028.s001.docx]

| **Table S1.** Pearson correlation of walking perceptions on each campus. | | | | | |
| --- | --- | --- | --- | --- | --- |
| Universities |  | Aesthetics | Security | Depression | Vitality |
| JNU | Aesthetics | 1.000 |  |  |  |
|  | Security | 0.623^***^ | 1.000 |  |  |
|  | Depression | -0.740^***^ | -0.582^***^ | 1.000 |  |
|  | Vitality | 0.242^***^ | 0.602^***^ | -0.375^***^ | 1.000 |
| ZJU | Aesthetics | 1.000 |  |  |  |
|  | Security | 0.564^***^ | 1.000 |  |  |
|  | Depression | -0.819^***^ | -0.587^***^ | 1.000 |  |
|  | Vitality | -0.041 | 0.439^***^ | -0.103^***^ | 1.000 |
| NJU | Aesthetics | 1.000 |  |  |  |
|  | Security | 0.674^***^ | 1.000 |  |  |
|  | Depression | -0.664^***^ | -0.571^***^ | 1.000 |  |
|  | Vitality | 0.475^***^ | 0.759^***^ | -0.497^***^ | 1.000 |
| ECNU | Aesthetics | 1.000 |  |  |  |
|  | Security | 0.648^***^ | 1.000 |  |  |
|  | Depression | -0.768^***^ | -0.530^***^ | 1.000 |  |
|  | Vitality | 0.295^***^ | 0.504^***^ | -0.356^***^ | 1.000 |

^*^*p* < 0.05; ^**^*p* < 0.01; ^***^*p* < 0.001.
