# Supplementary material for: Enhancing student-centered walking environments on university campuses through street view imagery and machine learning
Source: PLoS One. 2025 Apr 9;20(4):e0321028. doi: 10.1371/journal.pone.0321028 (PMC11981197; doi:10.1371/journal.pone.0321028)
Supplement: Attachment — Submitted filename: response.docx [file pone.0321028.s003.docx]

**Reviewer 1**

**Reviewer #1:** Enhancing student-centered walking environments on university campuses through street view imagery and machine learning
The authors' study on enhancing student-centered walking environments through street view imagery and machine learning is highly relevant and insightful. It effectively examines how campus environments impact students' academic performance and mental well-being by developing a deep learning framework. The use of Baidu Street View Images (BSVIs) and machine learning models like Random Forest and linear regression to predict perceptions of aesthetics, security, depression, and vitality offers a fresh approach to understanding campus spaces. The findings, which highlight how different visual elements such as vegetation, sky, and sidewalks influence student perceptions, provide valuable insights into designing more student-friendly walking environments.
This is a particularly interesting study because it links physical and human elements, reflecting the complexity of urban environments. The authors present a robust and consistent analysis. However, I have a few suggestions to further enhance the paper:
• The introduction rightly emphasizes the importance of campus walking perceptions, especially in China, where rapid educational expansion has led to the creation of new campuses. Well-designed walking spaces improve students' psychological health, encourage outdoor physical activity, and enhance academic performance. These spaces are crucial in fostering daily social interactions and are key to improving the overall quality of campus environments, as viewed through environmental psychology.

> Thank you for your comments.

• In addition to the authors' insights, recent studies have explored how urban design influences social behavior in various dimensions, such as well-being, health, and social relationships. I strongly recommend referring to "How Cities Influence Social Behavior" (MIT Press, 2024), which would provide greater depth and consistency to the arguments in this study.

> Thank you for your suggestion. We have added this content in the latest version. Please refer to Lines 96-114.

• The authors' use of recent technological advancements, such as crowdsourced services and geotagged images (Baidu Street View and Google Street View), is a notable strength of the paper. The results are timely and relevant. However, I suggest adding a section in the discussion to explore other emerging technologies. For instance, some recent studies have started evaluating the use of AI in the design and perception of cities, including aesthetics and aspects of well-being (e.g., "Leveraging Generative AI Models in Urban Science"). Expanding the discussion to include these advancements would provide a broader framework for the manuscript and position the research within a more comprehensive technological context.

> Thank you for your comments. We have incorporated a discussion on emerging technologies in the latest version. Please refer to Lines 439-442.

> Revised (Lines 439-442): *Emerging technologies, such as generative artificial intelligence (Generative AI), offer promising solutions by simulating temporal variations in street-view images, enabling more dynamic analyses of environmental impacts over time (Liu et al., 2024).*

**Reviewer 2**

**Reviewer #2:** Reviewer Comments to Author
This paper examines the relationship between the environmental features and four dimensions of walking perceptions among Chinese college students across four universities, utilizing street view imagery and machine learning technologies. It is an interesting and important exploratory study with the potential to contribute to the development of more walkable and healthier campus environmental design and planning.
However, several aspects require improvement to enhance the overall quality of the manuscript, particularly in the areas of the literature review, methods, and results. The authors are encouraged to avoid overinterpreting the results and drawing conclusions that extend beyond the scope of the study. Furthermore, the writing somewhat hinders the clarity and readability and requires improvement. Therefore, I recommend a major revision before the manuscript can be considered for publication. Please refer to my specific comments below for more details:

Title.
Please explain why your title is specific to student-centered walking environments, as there are many other campus users such as faculty, staff, and surrounding residents. Is it because you only recruit college students as volunteers? If so, please add more details on the volunteers in the Methods section.

> Thank you for raising this question. Our research is more focused on the student population. We have included additional information about the volunteers in the latest version. Please refer to Lines 281-286.

> Revised (Lines 281-286): *To ensure robust results, we recruited volunteers from diverse academic backgrounds to evaluate campus walking perceptions using a custom-designed computer platform. Volunteers were required to have at least one year of campus life experience, and to align with the study's target population, we primarily limited the participant pool to university students. Ultimately, 100 volunteers were retained, with 57.5% male and an average age of 21.30.*

Abstract.
1. The opening sentence should provide a research background that is closely related to the purpose of the study. The current version is too broad and lacks focus.
2. Please include more details about the study methodology. Specifically, mention the number of images analyzed, the involvement of volunteers, and relevant information about the campuses. This will give readers a clearer understanding of the study design and scope.

> Response to Q1 and Q2: We greatly appreciate your feedback. In the latest version, we have refined the abstract section to provide more details about our study.

> Revised (Lines 19-38): *Campus walking environments significantly influence college students' daily lives and shape their subjective perceptions. However, previous studies have been constrained by limited sample sizes and inefficient, time-consuming methodologies. To address these limitations, we developed a deep learning framework to evaluate campus walking perceptions across four universities in China's Yangtze River Delta region. Utilizing 15,596 Baidu Street View Images (BSVIs) and perceptual ratings from 100 volunteers across four dimensions—aesthetics, security, depression, and vitality—we employed four machine learning models to predict perceptual scores. Our results demonstrate that the Random Forest (RF) model outperformed others in predicting aesthetics, security, and vitality, while linear regression was most effective for depression. Spatial analysis revealed that perceptions of aesthetics, security, and vitality were concentrated in landmark areas and regions with high pedestrian flow. Multiple linear regression analysis indicated that buildings exhibited stronger correlations with depression (β = 0.112) compared to other perceptual aspects. Conversely, vegetation (β = 0.032) and meadow (β = 0.176) elements significantly enhanced aesthetics. This study offers actionable insights for optimizing campus walking environments from a student-centered perspective, emphasizing the importance of spatial design and visual elements in enhancing students' perceptual experiences.*

Introduction.
1. The introduction should clearly state why it is important to study the walking environment in higher education settings? What is the rationale behind focusing on this topic? Highlight the existing problems or challenges posed by inadequate walking environments and their impacts on students' daily academic experiences or health.
2. Consider combining paragraphs 2 and 3 to create a more cohesive and organized transition

> Response to Q1 and Q2: We sincerely appreciate your comments. We have thoroughly revised the introduction section.

> Revised (Lines 43-71): *The walking environment in higher education settings plays a critical role in shaping students' daily experiences, influencing their psychological well-being, physical health, and academic performance (De Vos et al., 2023; Gomez et al., 2021; Lee et al., 2020). Despite its significance, many university campuses face challenges related to inadequate walking environments, such as poor spatial design, limited accessibility, and insufficient integration of natural elements. These issues are particularly pressing in China, where the rapid expansion of educational opportunities has led to the proliferation of new campuses over the past two decades (Wang et al., 2021). Addressing these challenges is therefore urgent, as well-designed walking spaces enriched with natural elements have been shown to positively contribute to students' psychological well-being (Wang et al., 2018), promote outdoor physical activity (Dyment et al., 2007), and enhance academic performance (Hodson et al., 2017; Kweon et al., 2017).*

*Walking spaces on campuses encompass pathways that facilitate daily social interactions among students, defined by architectural surroundings and vegetation (Abusaada et al., 2021; Balsa-Barreiro et al., 2024). Environmental psychology views the walking experience as an interplay between the physical landscape and human behavior (Ewing et al., 2009; Zhang, Wang, et al., 2024), emphasizing the importance of diverse spatial attributes in shaping the subjective perception such as aesthetics, security, depression, and vitality (Dubey et al., 2016; Li et al., 2022; Zhang et al., 2018). Previous research has examined various dimensions of campus environments, including mental recovery (Akpinar, 2016), physical activity (Mårtensson et al., 2014), and academic performance (Hodson & Sander, 2017). Specific factors such as the green view index (GVI) (Yang et al., 2020) and sky view index (SVI) (Xi et al., 2012) have also been investigated. However, these isolated factors often fall short of capturing the complexity of students' perceptions. Moreover, traditional data collection methods, such as face-to-face interviews and questionnaires (Montello et al., 2017), are limited by small sample sizes, high costs, and time inefficiency, underscoring the need for more scalable and innovative approaches.*

Literature review
1. Consider incorporating more relevant, recent literature on each subtopic. For example, in section 2.1, as your study focuses on walking environments in university campuses and college students’ perception, please include references that specifically address these aspects. Please refer to the systematic review paper: Ding, Y., Lee, C., Chen, X., Song, Y., Newman, G., Lee, R., ... & Sohn, W. (2024). Exploring the association between campus environment of higher education and student health: A systematic review of findings and measures. Urban forestry & urban greening, 91, 128168.
2. Lines 122–124: Provide references to more recent literature to ensure the review remains current and reflective of recent advances in the field.

> Response to Q1 and Q2: We sincerely appreciate your comments. In the latest version, we have reorganized the literature review section to provide a more comprehensive understanding of the campus walking environment.

> Revised (Lines 96-114): *Well-designed walking spaces with natural environments on campuses have been shown to significantly impact students' well-being and academic performance (Lee & Shepley, 2020; Zhang, Wang, et al., 2024). These spaces can promote mental health (Wang et al., 2018), encourage outdoor physical exercise (Dyment & Bell, 2007), enhance academic performance (Li et al., 2016), and reduce campus violence (Fernandez, 2005). Recent studies have further emphasized the importance of campus walking environments in shaping students' perceptions and behaviors. For instance, Ding et al. (2024) conducted a systematic review highlighting that active transportation infrastructure, such as increased street connectivity and better walkability, is positively associated with students' physical activity and mental health. Additionally, several studies have identified specific features of walking spaces that significantly influence students' mental health and walkability perceptions. For example, the availability of greenery (De Vries et al., 2013), accessibility of facilities (Leyden et al., 2024), proximity to open spaces (Zhang, Sun, et al., 2024), aesthetic quality (Y. Zhao et al., 2017), and other walkability-related features have been shown to correlate strongly with students' psychological well-being and walking behavior. Hipp et al. (2016) found that perceived restorative qualities of walking spaces significantly improved students' quality of life, highlighting the importance of designing environments that foster relaxation and mental recovery.*

> Revised (Lines 141-151): *Recent advancements have deepened our understanding of human walking perceptions, encompassing dimensions such as security, comfort, and depression (Liu et al., 2021; Min et al., 2020). These insights not only inform urban planning and design (Shen et al., 2018) but also provide practical applications for understanding how walking space features shape human perception (Wang et al., 2022). However, research on campus walking perception using street-view images remains limited, with most studies relying on manually collected campus images (Q. Y. Liu et al., 2022; Tudorie et al., 2020; Wang et al., 2021). While these efforts have enhanced our understanding of human and student perceptions, challenges persist in extracting high-level information and achieving efficient pixel-wise classification of image features, underscoring the need for more advanced analytical approaches.*

3. Lines 156–157: Please specify why DeepLabV3+ was chosen for this study. Include justification for its suitability compared to other methods. In addition, fix the grammatical issues in this sentence to ensure clarity and correctness.

> Thank you very much for your comments. We have added further justification for selecting DeepLabV3+ as the foundational model.

> Revised (Lines 164-176): *To address this limitation, techniques like Atrous Spatial Pyramid Pooling (ASPP) have been integrated into architectures such as PSPNet (H. Zhao et al., 2017) and DeepLabV3 (Chen et al., 2017). These deep convolutional neural networks with pyramid pooling have shown superior performance in the semantic segmentation of urban street-view images (Gong et al., 2019; Tong et al., 2020). In this study, we employ DeepLabV3+ (Chen et al., 2018), an improved version of DeepLabV3, to model students' perceptions and extract high-level semantic information from campus street-view images. This model refines segmentation results by combining low-level and high-level features, enabling more precise boundary delineation. Compared to earlier models, DeepLabV3+ achieves a better balance between computational efficiency and segmentation accuracy, making it particularly suitable for analyzing complex campus environments. This approach aims to overcome the limitations of previous methods and provide a more comprehensive understanding of campus walking perceptions.*

Methods.
1. Line 164, why did you choose 50-meter intervals? Provide supporting evidence, references, or a rationale to justify this decision.

> Thank you very much for your comments. A 50-meter interval allows for a more comprehensive capture of environmental information and ensures the continuity of visual features at a spatial scale.

> Revised (Lines 218-222): *To ensure comprehensive coverage, we employed ArcGIS software to generate sampling points at 50-meter intervals along campus road networks, sourced from the OpenStreetMap (OSM) platform (Haklay et al., 2008). This interval was chosen to balance the capture of detailed environmental information with the spatial continuity of visual features (Ito et al., 2024; Ma et al., 2024; Zhao et al., 2023).*

2. Line 168, please provide additional details about the volunteers. Are they college students, professionals, or experts in the field? What qualifications make them suitable for image evaluations? Were there any inclusion or exclusion criteria.

> Thank you very much for your comments. We have added a more detailed description of the screening process in Section 3.4 of the Methods.

> Revised (Lines 283-293): *Volunteers were required to have at least one year of campus life experience, and to align with the study's target population, we primarily limited the participant pool to university students. Ultimately, 100 volunteers were retained, with 57.5% male and an average age of 21.30. Volunteers rated four perceptual dimensions on a 1-5 scale for a 20% subset (3,119 images) of the total street-view image dataset across four campuses, selected using a stratified random sampling method. Each image was evaluated by at least 50 volunteers, and the mean score was calculated. To ensure the reliability of the results, the standard deviation (std) of the ratings was monitored; if the std exceeded a predefined threshold (e.g., >1.5), additional ratings were collected until the std fell within an acceptable range. This approach minimized potential bias and enhanced the generalizability of the findings.*

3. Lines 211-212, please specify how you cleaned up the data and excluded invalid images. What criteria were used to determine invalid images, and what steps were taken to ensure data quality and consistency during preprocessing?

> Thank you very much for your comments. In the revised version, we have added details regarding the street view image screening process, as well as the measures taken to mitigate potential biases in the results caused by variations in brightness, color, and size among the images.

> Revised (Lines 218-222): *To ensure high data quality, we conducted rigorous data cleaning, removing invalid entries such as images with poor lighting, suboptimal viewing angles, or duplicates. The final dataset comprises 15,596 valid images across four directional views from JNU, ZJU, NJU, and ECNU. A detailed statistical breakdown of image collection for each campus is presented in Table 2. To minimize potential biases in the analysis, we used Python tools to uniformly adjust the brightness, color balance, and dimensions of all BSVIs.*

4. Lines 256-257, is a participant pool of 50 individuals sufficient to generalize the perception scores across a dataset of 15,596 images, particularly considering the dataset likely includes diverse images with varying levels of environmental perceptions? Address potential limitations due to the small participant pool and justify how you ensure the perception scores are not biased.

> Thank you very much for your comments. In the latest version, we have increased the number of volunteers and implemented multiple rounds of image scoring to minimize potential biases in the results.

> Revised (Lines 283-288): *Volunteers were required to have at least one year of campus life experience, and to align with the study's target population, we primarily limited the participant pool to university students. Ultimately, 100 volunteers were retained, with 57.5% male and an average age of 21.30. Volunteers rated four perceptual dimensions on a 1-5 scale for a 20% subset (3,119 images) of the total street-view image dataset across four campuses, selected using a stratified random sampling method.*

5. Lines 259, please provide more details on the question items utilized to measure four perceptual dimensions. In addition, regarding the measurement of depression, this term often refers to a chronic mental health condition, and I was concerned about its validity as a momentary measure. Please conduct further research on depression measurement to ensure the measurement approach is justified and appropriate.

> Thank you very much for your comments. We have provided additional details regarding the question items used to measure the four perceptual dimensions. Specifically, we have clarified that our study focuses on the instantaneous feeling of depression, and we have referenced several papers to offer a more comprehensive explanation.

> Revised (Lines 274-280): *For depression perception, we adopted a momentary measure of environmental depression, which reflects the transient emotional response evoked by the visual characteristics of a space rather than a clinical diagnosis of chronic mental health conditions. This approach aligns with previous studies that have successfully used momentary measures to assess the emotional impact of built environments (Ito et al., 2024; Zhang et al., 2018). For detailed questions related to each indicator, please refer to Table 3.*

| ***Table 3.*** *The question items utilized to measure four perceptual dimensions.* | | |
| --- | --- | --- |
| *Indicator* | *Question* | *Measurement (Likert scale)* |
| *aesthetics* | *"How visually appealing do you find this campus environment?"* | *1-5* |
| *security* | *"How safe do you feel in this campus environment?"* | *1-5* |
| *depression* | *"How depressing or gloomy does this campus environment make you feel?"* | *1-5* |
| *vitality* | *"How lively or vibrant do you find this campus environment?"* | *1-5* |
| *Note: 1 means strongly disagree, 5 means strongly agree.* | | |

Results.
1. Lines 277-279, please justify this sentence by providing valid and relevant references to support the claim.

> Thank you very much for your comments. We have removed the unreasonable descriptions and reorganized the paragraph for better clarity.

> Revised (Lines 306-312): *Table 4 summarizes the prediction performance of four machine learning models across four perceptual dimensions. The evaluation metrics, R² and MAE, reveal that RF outperformed other models in predicting aesthetics (R² = 0.81, MAE = 1.69), security (R² = 0.82, MAE = 1.93), and vitality (R² = 0.83, MAE = 1.82). In contrast, LR achieved the best performance for depression prediction (R² = 0.79, MAE = 1.90). Based on these results, RF and LR were selected as the optimal models for predicting perceptual scores across the entire dataset of 15,596 valid campus street-view images.*

2. Lines 327-329, please specify how perception scores were linked to each feature to create Figure 7. From the methods described, it seems volunteers evaluated perception scores for each image rather than for individual features. If this is the case, explain the process used to associate perception scores with specific features.

> Thank you very much for your comments. After careful consideration, we have decided to remove this section. Please refer to Lines 314-379.

3. Line 335-337, consider moving this sentence to the Discussion section to maintain focus in the Results section on interpreting data.

> Thank you very much for your comments. Indeed, the results section is not the most appropriate place for extensive discussion. In accordance with your suggestion, we have relocated this content to the discussion section. Please refer to Lines 314-379.

4. For table 7, please refine the table following the APA format, including key statistical information such as 95% confidence intervals and R-squared values. Also specify the units of the measurement for the 10 features in this analysis.

> Thank you for pointing this out. In the latest version, we have added the 95% CI and R-squared values, and clarified that the unit of measurement for the features is % (i.e., the visual proportion of the features). Please refer to Lines 378-379.

| **Table 7.** Results of regression analysis for the visual features and perception scores. | | | | | | | | |
| --- | --- | --- | --- | --- | --- | --- | --- | --- |
| Standardization features (%) | Aesthetics  (*Beta*) | 95% CI | Security  (*Beta*) | 95% CI | Depression  (*Beta*) | 95% CI | Vitality  (*Beta*) | 95% CI |
| Vegetation | 0.032*** | [0.031, 0.034] | 0.019*** | [0.017, 0.021] | 0.097*** | [0.096, 0.099] | 0.022*** | [0.021, 0.024] |
| Sky | 0.059*** | [0.057, 0.062] | 0.043*** | [0.040, 0.046] | 0.002 | [-0.000, 0.005] | 0.055*** | [0.052, 0.058] |
| Road | 0.112*** | [0.108, 0.117] | 0.136*** | [0.130, 0.143] | 0.017*** | [0.012, 0.023] | 0.119*** | [0.114, 0.125] |
| Building | 0.013*** | [0.011, 0.015] | 0.044*** | [0.040, 0.047] | 0.112*** | [0.109, 0.115] | 0.038*** | [0.036, -0.042] |
| Meadow | 0.176*** | [0.172, 0.182] | 0.113*** | [0.106, 0.120] | 0.016*** | [0.010, 0.023] | 0.023*** | [0.017, 0.030] |
| Sidewalk | 0.154*** | [0.147, 0.161] | 0.147*** | [0.136, 0.157] | -0.061*** | [-0.070, -0.052] | 0.092*** | [0.083, 0.102] |
| Fence | 0.013*** | [0.009, 0.017] | 0.026*** | [0.021, 0.033] | 0.095*** | [0.090, 0.101] | 0.041*** | [0.035, -0.046] |
| Wall | 0.027*** | [0.019, 0.035] | 0.037* | [0.026, 0.049] | 0.085*** | [0.076, 0.095] | 0.006 | [-0.005, 0.016] |
| Car | -0.052*** | [-0.065, -0.039] | 0.018* | [0.001, 0.037] | 0.255*** | [0.239, 0.271] | 0.239*** | [0.223, 0.257] |
| Person | 0.116*** | [0.091, 0.143] | 0.236*** | [0.199, 0.273] | 0.020 | [-0.012, 0.052] | 0.527*** | [0.493, 0.563] |
| R² | 0.622 |  | 0.583 |  | 0.558 |  | 0.601 |  |

Note: 95% CI = 95% confidence interval; Significance levels: ***p < 0.001, **p < 0.01, *p < 0.05.

5. Table 7 indicates that the vegetation was negatively associated with aesthetics, security and vitality, and positively related to depression. This is kind of conflating with previous studies. The interpretation of these results (Lines 407–409) appears abrupt, ambiguous, and overly intuitive. How can it be determined that vegetation is "excessive and dense" rather than "well-maintained and healthy"? Provide evidence or further analysis to support this statement. If this claim is speculative, acknowledge the uncertainty and suggest possible explanations in the Discussion section.

> Thank you for pointing this out. We have reanalyzed the results. Please refer to Lines 355-379.

> Revised (Lines 355-371): *To explore the relationships between the top 10 visual features and four walking perceptual dimensions, we conducted a multiple linear regression analysis. Table 7 presents the standardized beta coefficients, highlighting both positive and negative correlations across the dimensions. Person showed the strongest positive correlation with vitality (β = 0.527, p < 0.001) and security (β = 0.236, p < 0.001), while car features exhibited the strongest negative association with aesthetics (β = -0.052, p < 0.001). The meadow (β = 0.176, p < 0.001) and sidewalk (β = 0.154, p < 0.001) features demonstrated substantial positive correlations with aesthetics. Road features consistently showed positive correlations across all dimensions, including aesthetics (β = 0.112, p < 0.001), security (β = 0.136, p < 0.001), and vitality (β = 0.119, p < 0.001). Vegetation showed a positive correlation with other dimensions, albeit with smaller coefficients. Cars showed mixed effects, negatively impacting aesthetics (β = -0.052, p < 0.001) while strongly positively associating with both depression (β = 0.255, p < 0.001) and vitality (β = 0.239, p < 0.001). Buildings demonstrated a stronger positive correlation with depression (β = 0.112, p < 0.001) compared to other dimensions. Wall features showed non-significant effects on vitality (p > 0.05), while significantly correlating with other dimensions, particularly depression (β = 0.085, p < 0.001).*

> Revised (Lines 372-371): *The results suggest that natural elements, such as meadows and open skies, play a crucial role in enhancing positive perceptions, while built features like walls and dense vegetation may have adverse effects. These findings provide actionable insights for urban planners and designers aiming to create more inviting and psychologically supportive campus environments.*

Discussion/Conclusion.
1. For limitations, it is important to highlight that this is an exploratory study based on data from only four schools, which may limit external validity and generalizability.

> Thank you for your suggestion. We have emphasized this point in the study limitations.

> Revised (Lines 431-433): *Our study has three main limitations. First, the findings are based on data from only four campuses, which may constrain the external validity and generalizability of the results.*

2. Ensure that the discussion and conclusion are closely aligned with the study’s actual findings. Please avoid over interpretation (i.e. discussion about excessive vegetation) and ambitious conclusions related to theory development (Line 430).

> Thank you for raising this question. In the latest version, we have focused more on discussing the research results and avoided excessive discussion of vegetation-related content.

> Revised (Lines 396-406): *Mapping intangible perceptions revealed significant spatial heterogeneity across campus walking environments. Positive perceptions, such as aesthetics, security, and vitality, often concentrated around campus landmarks, including main gates, libraries, and waterscapes. For instance, natural elements around waterscapes at JNU (Fig. 5a1) enhanced aesthetic quality (Kaplan & Kaplan, 1989), while open spaces with expansive views and minimal visual obstructions allowed for more sunlight and sky exposure, encouraging prolonged outdoor stays and public activities (Horvat et al., 2023). Conversely, negative perceptions, such as depression, were more prevalent in peripheral areas and dense building zones, such as dormitories. Notably, administrative, living, and teaching areas exhibited high perceptual agreement, while ecological recreation areas at ZJU showed inconsistent results.*

> Revised (Lines 407-416): *To better understand the relationship between walking space features and perceptual dimensions (aesthetics, security, depression, and vitality), we conducted multiple regression analyses (Section 4.3.2). The top 10 environmental features provide actionable insights for campus design. Specifically, maximizing open spaces with expansive views and minimal visual obstructions (e.g., walls, fences) were linked to higher perceptions of aesthetics and vitality. These areas allow for more sunlight and sky exposure, which can prolong outdoor stays and encourage public activities (Q. Y. Liu et al., 2022; Wang et al., 2021). Campus planners should prioritize the creation and maintenance of such spaces, particularly around key landmarks like libraries and waterscapes, to enhance student well-being and social interaction.*

3. Expand on the implications for future research and campus design guidelines. Discuss how these findings can inform practical strategies for creating more walkable and health-promoting campuses.

> Thank you for your suggestion. Based on the research findings, we have specifically proposed future research directions and campus design guidelines.

> Revised (Lines 417-430): *Furthermore, addressing building density is critical. High-density building areas, such as dormitory complexes, were associated with negative perceptions like depression. To mitigate this, future campus designs should incorporate more open spaces, greenery, and natural light in densely built areas. Transitional spaces between buildings, such as courtyards or plazas, can help break up monotony and improve perceptual quality (G. Liu et al., 2022). Additionally, enhancing safety through design is another key consideration. Features like well-lit sidewalks, clear sightlines, and visible landmarks were positively associated with security perceptions. Campus planners should prioritize these elements, particularly in peripheral or less-trafficked areas, to create a sense of safety and encourage nighttime use of outdoor spaces (Cheng et al., 2020). By incorporating these strategies, campus designers can create environments that not only support walking but also enhance mental health, social interaction, and overall well-being. These findings provide practical guidance for creating more inclusive, vibrant, and restorative campus spaces.*

Other issues
1. Please proofread the manuscript thoroughly to correct all grammatical errors and typos, enhancing its readability.

> Thank you for your suggestion. We have thoroughly proofread the manuscript.
