# Supplementary material for: Enhancing student-centered walking environments on university campuses through street view imagery and machine learning
Source: PLoS One. 2025 Apr 9;20(4):e0321028. doi: 10.1371/journal.pone.0321028 (PMC11981197; doi:10.1371/journal.pone.0321028)
Supplement: Attachment — Submitted filename: response_r2.docx [file pone.0321028.s004.docx]

**Reviewer 1**

Reviewer #1: Dear Authors,

I would like to commend you for the comprehensive and thoughtful revisions made to your manuscript. Your responses effectively addressed all of my concerns, and the improvements have enhanced the overall clarity and impact of your work.

Given these significant enhancements, I am pleased to recommend your manuscript for publication. Congratulations on this achievement, and I wish you success in your future research endeavors.

Best regards, the Ass. Editor

> Thank you very much for your review comments.

**Reviewer 2**

Reviewer #2: The authors have effectively addressed all reviewer comments and revised the paper in line with the suggestions. The new version has improved significantly in both presentation and content, now appearing much more incisive overall.

Some minor issues need to be addressed before publication:

1. Replace “Person correlation” with “Pearson correlation” in Figure 1.

> Thank you for your comments. We have fixed this error, please see Line 189.

2. Clarify where Appendix Table S1 is cited in the main text.

> Thank you for your comments. We have revised this problem.

> Revised (Lines 324-326): *Pearson correlation analysis result of four perceptual dimensions predicted score among each campus shown in Appendix Table S1.*
